# Distribution of the invasive plant species *Heracleum sosnowskyi* Manden. in the Komi Republic (Russia)

**DOI:** 10.3897/phytokeys.77.11186

**Published:** 2017-03-09

**Authors:** Ivan Chadin, Igor Dalke, Ilya Zakhozhiy, Ruslan Malyshev, Elena Madi, Olga Kuzivanova, Dmitrii Kirillov, Vladimir Elsakov

**Affiliations:** 1 Institute of Biology of Komi Scientific Centre of the Ural Branch of the Russian Academy of Sciences, Kommunisticheskaya, 28, 167982, Syktyvkar, Komi Republic, Russian Federation

**Keywords:** Occurrence, human observation, *Heracleum sosnowskyi*, hogweed, invasive, geotagged photographs, Komi Republic, European North-East Russia

## Abstract

Occurrences of the invasive plant species *Heracleum sosnowskyi* Manden. in the Komi Republic (northeastern part of European Russia) were recorded and published in the Global Biodiversity Information Facility (GBIF http://www.gbif.org) using the RIVR information system (http://ib.komisc.ru/add/rivr/en). RIVR stands for “Rasprostranenie Invasionnyh Vidov Rastenij” [Occurrence of Invasion Plant Species]. This citizen science project aims at collecting occurrence data about invasive plant species with the help of citizen scientists. Information can be added by any user after a simple registration (concept) process. However, the data published in GBIF are provided only by professional scientists. The total study area is approximately 19,000 km^2^. The GBIF resource contains 10894 *Heracleum sosnowskyi* occurrence points, each with their geographical coordinates and photographs of the plants in the locus of growth. The preliminary results of species distribution modelling on the territory of European North-East Russia presented.

## Project details

### Project title

“Ecophysiological modelling of invasive plant species distribution. The case of *Heracleum sosnowskyi* in the taiga zone of the European part of Russia”

### Funding

The project was supported by a grant of the Russian Foundation for Basic Research and the Government of Komi Republic (Project No 16-44-110694).

### Study area description

The Komi Republic is located in the north-east of the Russian Plain and the western slopes of the northern Ural Mountains. It is a large and an important biogeographic boundary that separates the flora and fauna of two continents – Europe and Asia.

On the plain territory of the Komi Republic, a pronounced latitudinal-nature zonation occurs. The extreme north-east is taken by a subzone of the southern tundra. The forest-tundra is a transition zone between the tundra and taiga. In the Pechora Province, it has a width of 100–120 km forming the southern periphery of the territory that has the Bolshezemelskaya tundra. The main type of vegetation in the Republic of Komi is the boreal (taiga) forest. The taiga zone is divided into the following subzones: Extreme northern, Northern, Middle, and Southern. The eastern edge of the Republic in occupied by the Ural Mountains, where altitudinal zonation occurs with distinct Mountain forest, Alpine tundra, and Cold deserts zones (Gorchakovskij 1975).

A large part of the republic has a climate similar to that of the Atlantic-Arctic region with a cold temperate (boreal) climate (Brattsev et al. 1997). The territory is a zone of excessive moisture, widespread marshes, and wetlands. The annual precipitation exceeds the evaporation and decreases from south to north, from 700 to 550 mm. A significant difference in the climate is observed across the length of the republic from south to north and from west to east. The duration of the winter in the south of the republic is 170–180 days and that in the north is 230–250 days. The average temperature in January (the coldest month) in the south is 15 °C whereas that in the north-east is –22 °C. Summers are short and warm; the average temperature in July (the warmest month) is approximately 10°C in the north-east and 17°C in the south. The prevailing wind directions in winter are south and south-west, and north in summer. The monthly average wind speed in the taiga zone is 3–4 m/s and that in the tundra area is 6.5 m/s.

Biological diversity of the Komi Republic region includes 929 fungi, 1217 vascular plants, 653 moss, 1020 lichen, 2,000 algae, more than 3,500 arachnid, more than 6,000 insect, 50 fish, six amphibian, five reptile, 265 bird, and 57 mammal species. There are 237 forest, floristic, meadow, marsh, ichthyological, ornithological, and geological reserves and natural monuments on the territory of Komi. The Pechora-Ilych State Reserve and the Yugyd Va National Park occupy 13.5% of the total territory of the republic (Ponomarev and Tatarinov 2012).

### Design description

The project design combines an experimental approach and analysis of results of the observations. The responses of *Heracleum sosnowskyi* plants to the changes in the abiotic environmental parameters were obtained by instrumental measurements of the morphological and physiological parameters (including CO_2_/H_2_O gas exchange, chlorophyll fluorescence, and heat dissipation) in the plants grown in climatic chambers and experimental plots. The data of the optimal and critical values of the environmental factors (heat, light, rainfall, and soil) required for the survival and reproduction of the plants were used for a joint analysis along with the geographically referenced data of these factors. The results were arranged in a raster map showing the potential areas of *Heracleum sosnowskyi*. The resulting map was verified by a direct comparison with the data of the field observations of the habitats of this species and with the correlation simulation of their geographical distribution.

### Data published through

GBIF: http://ib.komisc.ru:8088/ipt/resource?r=heraclueum_occurrence

## Taxonomic coverage

### General taxonomic coverage description

The resource contains occurrence data only for one species – *Heracleum sosnowskyi* Manden.

### Taxonomic ranks

Kingdom: Plantae

Phylum: Tracheophyta

Class: Magnoliopsida

Order: Apiales

Family: Apiaceae

Genus: *Heracleum*

Species: *Heracleum sosnowskyi*

Common names: Sosnowsky’s hogweed, plants, vascular plants, flowering plants, carrot family, hogweed

## Spatial coverage

### General spatial coverage

The geographical coverage is essentially limited to the Komi Republic territory located in the European part of Russia. Currently, all populations of *Heracleum sosnowskyi* in this area are invasive. This species was introduced into this region in the second half of the 20^th^ century as a forage crop. Since 2012 varieties of this species are excluded from the register of the breeding achievements of the Russian Federation (Official bulletin 2012; http://gossort.com/bullets/pdf/bull_176.pdf) . This species is also included in the “specialised catalogue of weeds” (Information letter 2015; http://antibor.ru/sites/526a0b00d7e1e49744000002/assets/56fa0dcdd7e1e4c087062929/pismo1-2.jpg).

### Coordinates

59°22.48'N and 66°7.12'N Latitude; 48°56.24'E and 60°20.24'E Longitude

### Temporal coverage

28 July 2012 - 23 August 2016

## Methods

### Method description

Photographs of plants were taken using consumer cameras. Videos were recorded with a Car DVR Camera (video 1280×960 pixels at 30 frames/second), mounted on the car windshield (height from the road surface was 170 cm). The survey was conducted at speeds of 60–90 km/h. The GPS track was simultaneously recorded with GPS navigators. The time on the cameras and video recorders were synchronised with the time displayed on the GPS navigation device.

All the images were geotagged by a GPS track log with “GPS Correlate” software (v 1.6.1, https://github.com/freefoote/gpscorrelate) according to the methods described in the OpenStreetMap Project documentation (Geotagging Source Photos 2016; http://wiki.openstreetmap.org/wiki/Geotagging_Source_Photos). The video files were broken into frames (one frame per second) and the frames were saved as “jpeg” files with the program FFmpeg (v 3.1.4 http://www.ffmpeg.org) followed by geotagging of these files similar to that of the photographs. The array of images was hand sorted into two groups: images that contained *Heracleum sosnowskyi* plants and images without these plants. The coordinates of the photographs obtained from a Car DVR Camera were corrected in the Quantum GIS Geographic Information System (QGIS) program (v 2.16.3 http://www.qgis.org, QGIS Development Team 2016) by shifting the group of points on the side of the road. All geotagged *Heracleum sosnowskyi* images were uploaded to the online database “Occurrence of invasive plant species *Heracleum sosnowskyi* Manden.” (RIVR 2016).

### Study extent description

**Figure 1.**
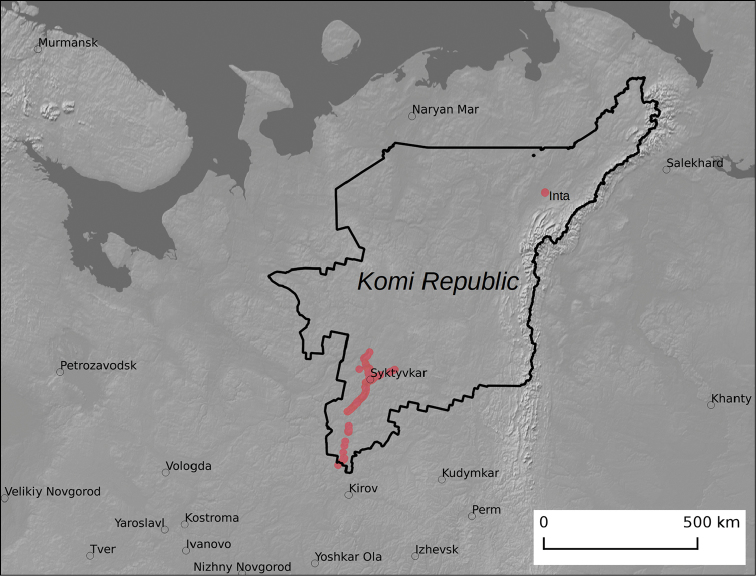
Study area. Red points indicate occurrences of *Heracleum sosnowskyi* described in the data paper.

The occurrence data of *Heracleum sosnowskyi* were collected from an area of approximately 19, 000 km^2^ (Figure 1). Most of the data were collected from the capital area of Komi, Syktyvkar (61°39.95'N, 50°49.53'E) as well as along the roads at a distance of 300 km from Syktyvkar, the directions of which coincide with the flow direction of the major rivers Vychegda and Sysola belonging to the Northern Dvina basin. A separate cluster of the data was collected from a 664 km (orthodromic) distance in the territory and suburb of Inta city, located near the Arctic Circle (66° 1.87’N, 60° 8.72’E). A pronounced sampling bias should be considered before using the data for the species distribution modelling. Data were collected close to the settlements or the roads connecting them, which is a travel time bias (Fourcade et al. 2014). In the case of *Heracleum sosnowskyi*, such a sampling bias may coincide with the actual factors determining the dispersal of the plants of this species. In most cases, roadsides are the optimal habitats for this species as they are open and well-lighted with adequate moisture due to the roadside drainage systems. Moreover, the air flow creates favourable conditions for the spread of the plants.

### Sampling description

The occurrence data consist of the presence data only. Two methods were used for the creation of occurrence records, which include the data collection along transects (7130 points) and mapping of *Heracleum sosnowskyi* boundaries that were later converted to regular points sample (3764 points). The regular points sample coordinates were generated using the QGIS Desktop software (v 2.16.3). The points were created with a 25 m point spacing within polygon layers that indicated the *Heracleum sosnowskyi* population boundaries. The occurrences were labelled with a tag “Generated Regular Sample” written in the “occurrence remarks” field. The “associated media” field contained the URL of the locality map showing the generated point pattern with the scale bar and the north end on top of the map.

Data along transects were collected by recording a video of *Heracleum sosnowskyi* plants growing along the roadsides and by taking photographs in the direction perpendicular to the road at a distance of up to 5 km.

### Quality control description

The published data collected by professional scientists with sustainable skills for the identification of *Heracleum sosnowskyi* and its differences from other similar species in its habitats were published in GBIF whereas that collected by volunteers were accumulated in the RIVR system. Before publication, data were checked for gross errors in georeferencing by visual inspection of the overlay points on the map with the borders of Russian regions in OpenStreet in the QGIS Desktop.

The presence of duplicate records was checked by running a special SQL script. The records were counted as duplicated if three fields were the same: the coordinates, the date of the event, and the file name of the photograph. For many data points (1080 of 10894 points, 10%), the same dates and coordinates were detected; however, they presented a series of photographs (2 to 13). These data were saved in the system as they could be of interest for the assessment of the landscape and the evaluation of plants in the *Heracleum sosnowskyi* habitat.

### Species distribution modelling

The described dataset was used for *Heracleum sosnowskyi* species distribution modelling (SDM). The SDM was performed for two plots. Plot 1 was a rectangular, limited by latitudes: 61.0088°N, 62.1387°N and longitudes: 49.5013°E, 51.5941°E. The area of Plot 1 was 9 180 km^2^. The Plot 2 was a rectangular, limited by latitudes: 57.0000°N, 70.0000°N, 42.0000°E, 68.0000°E. The area of Plot 2 was 1 857 586 km^2^. All coordinates were given in the WGS84 projection (EPSG: 4326).

Two groups of predictors were used. Group 1: the state of the earth’s surface, with a spatial resolution of 1 second (≈ 30 m) per pixel (data was collected for Plot 1only): VEG = vegetation cover map derived from classification of satellite images (20 classes); ROAD = proximity map to the nearest road; AGRO = proximity map to the nearest borders of agricultural areas. Group 2: bioclimatic variables are derived from the monthly temperature and rainfall values obtained from WorldClim (Hijmans et al. 2005; http://www.worldclim.org/bioclim) with resolution of 30 second (≈ 1000 m) per pixel (data was collected for Plot 1 and Plot 2): BIO1 = Annual Mean Temperature; BIO2 = Mean Diurnal Range; BIO3 = Isothermality; BIO4 = Temperature Seasonality; BIO5 = Max Temperature of Warmest Month; BIO6 = Min Temperature of Coldest Month; BIO7 = Temperature Annual Range (BIO5-BIO6); BIO8 = Mean Temperature of Wettest Quarter; BIO9 = Mean Temperature of Driest Quarter; BIO10 = Mean Temperature of Warmest Quarter; BIO11 = Mean Temperature of Coldest Quarter; BIO12 = Annual Precipitation; BIO13 = Precipitation of Wettest Month; BIO14 = Precipitation of Driest Month; BIO15 = Precipitation Seasonality (Coefficient of Variation); BIO16 = Precipitation of Wettest Quarter; BIO17 = Precipitation of Driest Quarter; BIO18 = Precipitation of Warmest Quarter; BIO19 = Precipitation of Coldest Quarter.

All data were obtained from open sources, either directly or as a result of raw data processing in geographic information systems. The rights to use the Komi Republic agriculture area map were acquired under a license agreement with the State Organization “Syktyvkar Agrochemical Service Station”.

The presence data of *Heracleum sosnowskyi* occurrences were obtained as a random sample of GBIF dataset described in this article. Five hundred randomly chosen presence points were taken for modelling at Plot 1 and 1000 points for modelling at Plot 2. Furthermore, 500 (for Plot 1) and 1000 (for Plot 2) randomly distributed points were used as a background point.

SDM was performed with generalized linear multiple regression model in R (R Core Team 2014) with dismo package (Hijmans et al. 2017).

**Figure 2.**
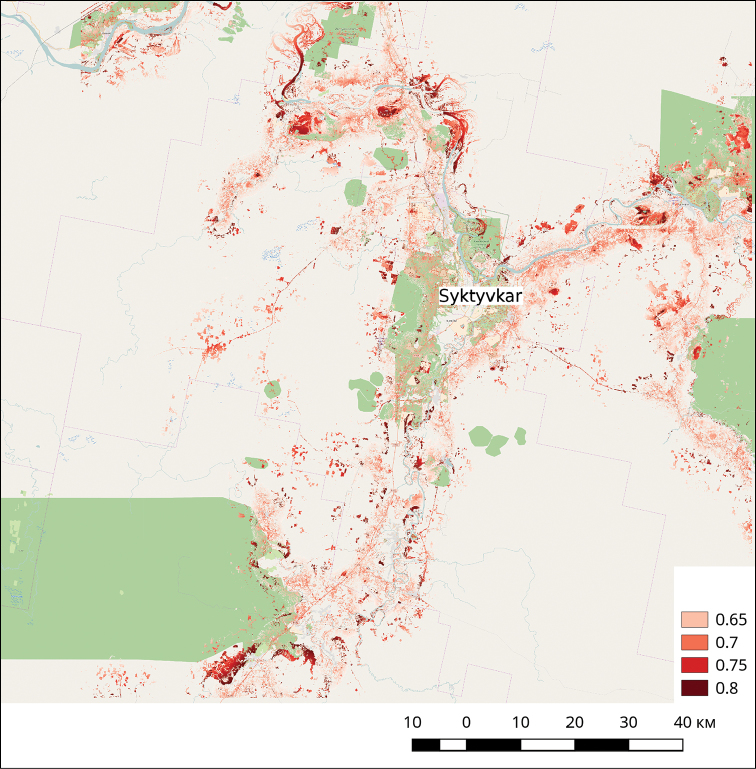
The prediction map of *Heracleum sosnowskyi* habitats prepared with the species distribution model based on vegetation cover map, nearest road proximity map, proximity map to the borders of agricultural areas. The colour scale shows the probability *Heracleum sosnowskyi* presence.

Model fitting with the predictors VEG, ROAD and AGRO showed statistically significant (p < 0.0001) relationship with the dependent variable (*Heracleum sosnowskyi* presence in the given point). ROC analysis showed that AUC value for the regression model was 0.92). These results were supported by field observations, invasion history and ways of *Heracleum sosnowskyi* seed dispersal. The plant occupies habitats with disturbed soil cover, spreading rapidly along roads, due to the transfer of seeds by air flow, avoids shaded and dry habitats (Fig. 2).

**Figure 3.**
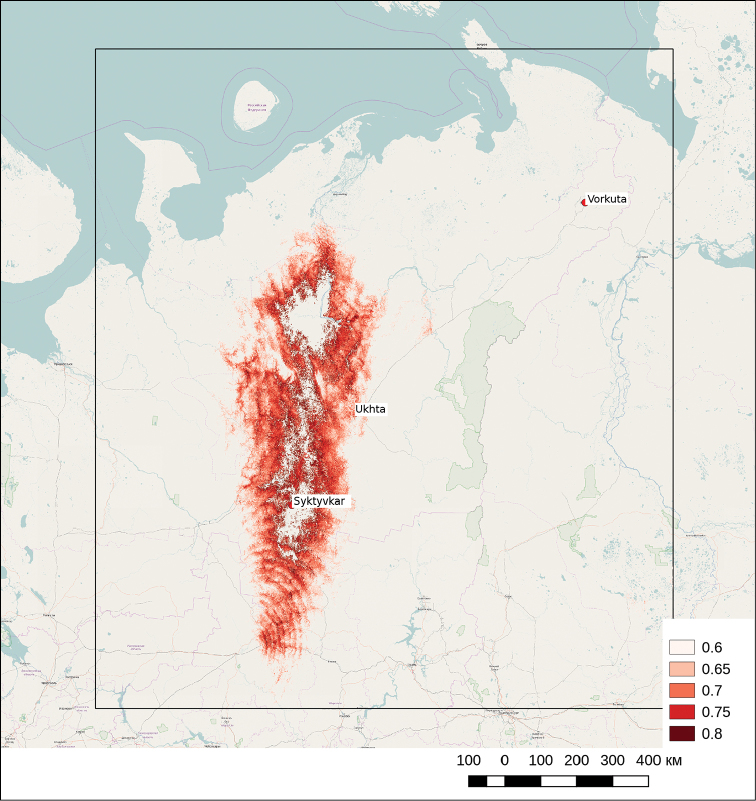
The prediction map of Heracleum sosnowskyi habitats prepared with the species distribution model based on bioclaimatic predictors. The borders of Plot 2 within which the model prediction was made. The colour scale shows the probability *Heracleum sosnowskyi* presence.

Model fitting at Plot 1 and Plot 2 with bioclimatic predictors revealed a statistically significant relationship with eight predictors: BIO2, BIO4, BIO5, BIO6, BIO7, BIO10, BIO12 and BIO17. The model with all these predictors showed AUC value 0.99. Prediction with the model obtained within Plot 2 allowed to identify the putative northern *Heracleum sosnowskyi* range boundary — 67.2000°N, within the borders of the valley of the Pechora river (Fig. 3). According to the model, the values of bioclimatic variables in the areas with maximum probability of *Heracleum sosnowskyi* presence were as follows (mean and standard deviation): BIO2: 8.3 ± 0.2 °C, BIO4: 112 ± 1 °C, BIO5: 21.2 ± 0.6 °C, BIO6: -21.9 ± 0.3 °C, BIO10: 3.6 ± 0.6 °C, BIO12: 567 ± 24 mm.

The presence of *Heracleum sosnowskyi* invasive plants in the northern forest-tundra subzone (66.0000°N) was confirmed by field observation on the territory of Inta city (Komi Republic). *Heracleum sosnowskyi* plants formed monostand and showed high enough seed productivity (up to 12 000 seeds per plant) in this area.

## Datasets

### Dataset description

**Object name**: Darwin Core Archive Occurrences of the invasive plant species *Heracleum sosnowskyi* Manden. in the Komi Republic (European North-East Russia)

**Character encoding**: UTF-8

**Format name**: Darwin Core Archive format

**Format version**: 1.0

**Distribution**: http://ib.komisc.ru:8088/ipt/archive.do?r=heraclueum_occurrence

**Publication date of data**: 2016-10-19

**Language**: English

**Licences of use**: This work is licensed under a Creative Commons Attribution (CC-BY) 4.0 License.

**Metadata language**: English

**Date of metadata creation**: 2016-09-07

**Hierarchy level**: Dataset
